# Fixed-Ratio combinations (Basal Insulin Plus GLP-1RA) in Type 2 diabetes. An analytical review of pivotal clinical trials

**DOI:** 10.1900/RDS.2023.19.14

**Published:** 2023-03-31

**Authors:** Hernando Vargas-Uricoechea, Juan Pablo Frias, Hernando David Vargas-Sierra

**Affiliations:** 1 Metabolic Diseases Study Group, Department of Internal Medicine, Universidad del Cauca, Popayán-Colombia,; 2 National Research Institute, USA,; 3 Metabolic Diseases Study Group, Department of Internal Medicine, Universidad del Cauca, Popayán-Colombia. Fellowship in Endocrinology, Diabetes and Metabolism, Universidad Pontificia Bolivariana, Medellín-Colombia.

**Keywords:** degludec, glargine, liraglutide, lixisenatide, fixedratio combination

## Abstract

In type 2 diabetes, therapeutic failure to the oral anti diabetics is frequent, the use of schemes with basal insulin or with multiple doses of insulin (basal insulin and short-acting insulins) are a widely accepted way to intensify therapy. The use of GLP-1 receptor agonists is another intensification strategy. The fixedratio combinations with molecules such as insulin degludec + liraglutide, and insulin glargine + lixisenatide have proven useful in intensifying treatment of individuals with type 2 diabetes. The purpose of this review was to evaluate and analyze the results of pivotal studies with both fixed-ratio combinations in individuals with type 2 diabetes, finding that, they are capable of achieving better glycemic control when compared with each of its components separately (with a lower risk of hypoglycemia vs basal insulin and lower risk of gastrointestinal adverse effects vs GLP-1 receptor agonists) in various clinical scenarios, especially in individuals who do not achieve control with oral antidiabetics or who do not achieve control with basal insulin (associated with oral antidiabetics) or in those under management with GLP-1RA plus oral antidiabetics.

## 1. Introduction

More than 90% of all types of diabetes mellitus (DM) is type 2 DM (T2DM), which is a heterogeneous group of pathologies, characterized by a state of chronic hyperglycemia and a consequent increase in the risk of microvascular and macrovascular complications, mortality and other associated comorbidities. Multiple genetic and environmental factors have been associated, in addition to a significant number of pathophysiological mechanisms, highlighting insulin resistance and progressive pancreatic β-cell failure [1- 4].

For the year 2021, the people with DM worldwide were calculated at 537 million, and a significant increase in this number is estimated, being approximately 643 and 783 million for the year 2030 and 2045, respectively [5,6].

A good glycemic control has been associated with a significant reduction in the risk of long-term complications, and in this sense, the intensification of treatment is essential to achieve glycemic control and potentially reduce these complications [7,8].

Despite the medications available for the treatment, there is still a significant number of individuals who do not have adequate metabolic control. The international recommendations for the treatment of T2DM coincide in an individualized strategy according to risk and cardiovascular and/or renal involvement, and among the intensification strategies (additional to oral anti diabetics –OADs–) stand out: *basal insulins (BIs), rapid-acting insulins, and GLP-1 receptor agonists (GLP-1RAs)* [9-12].

The use of BIs together with GLP-1RAs has been shown to be effective in achieving A1c, fasting plasma glucose (FPG) and postprandial glucose (PPG) goals, with less weight gain and less risk of hypoglycemia. Additionally, most of the GLP-1RAs have shown a reduction in the risk of cardiovascular and renal outcomes, which means that when they are used concomitantly with BIs, the beneficial effects provided by each molecule can be preserved [13-16].

Recently, the introduction of the so-called fixedratio combinations (FRC) which combine BI + GLP- 1RA (in the same application device) have changed the perspective of parenteral management in T2DM.

The purpose of this review is to describe and analyze the results of published trials with the two currently available FRCs: insulin degludec U-100 (iDeg) + liraglutide (Lira) –iDegLira– and, insulin glargine U-100 (iGlar) + lixisenatide (Lixi) –iGlarLixi–.

## 2. Methods

### 2.1 Data selection

The following electronic databases were searched: PubMed and MEDLINE, Scopus, BIOSIS, ClinicalTrials.gov, Embase, Cochrane, Google Scholar, and Springer Online Archives Collection, from January 2010 to august 2022, using the terms “insulin,” “glargine”, “degludec”, “basal insulin”, “GLP-1RA”, “Fixed-ratio combination” with the term “type 2 diabetes.” Articles resulting from these searches and relevant references cited in those articles were examined. Only pivotal randomized clinical trials (RCTs) published in English were included in this review.

### 2.2 Rationale for clinical use of FRCs in individuals with T2DM

The pathophysiological component of T2DM indicates that, over time, the probability of requiring different schemes with insulin increases. The most frequent of the schemes is with BIs, nevertheless, if metabolic control is not achieved (upon reaching a daily dose of BI of 0.5 U/kg/day), then it is necessary to intensify the therapy. Among the recommended guidelines, the use of rapid-acting insulins (in multiple insulin dose schemes) stand out; however, this type of treatment significantly increases body weight and the risk of hypoglycemia. The use of GLP-1RAs is another intensification strategy, since in addition to its efficacy in glycemic control, it also causes weight loss and a low risk of hypoglycemia, which makes it an attractive option for intensification [17-20].

The main reason that justifies the use of FRCs is due to the complementary mechanism of action of both components; for example, BIs inhibits the hepatic glucose production and increases glucose uptake in liver, muscle, and adipose tissue. Additionally, BIs complements the action of endogenous insulin and, therefore, can protect β-cells, improving prandial insulin response [21-23].

Otherwise, GLP-1RAs act by slowing gastric emptying, in addition to decreasing glucagon secretion and, therefore, PPG (especially with lixisenatide) and increasing insulin secretion, all together with a decrease in food intake caloric (by increasing the feeling of satiety); further, the GLP-1RAs may improve pancreatic function by increasing β-cell proliferation and inhibiting β-cell apoptosis [21-23].

Consequently, the addition of a GLP-1RAs in individuals with some insulin requirement, would allow the use of a lower dose of BIs, with an additional benefit on weight loss, and a lower risk of hypoglycemia [21-25].

### 2.3 RCTs assessing the efficacy and safety of iGlarLixi

The efficacy and safety of iGlarLixi was evaluated in the LixiLan program and in the SoliMix trial. The LixiLan program are three RCTs (in three different populations of participants with T2DM): *subjects not controlled with OADs (LixiLan-O), subjects not controlled with BIs (LixiLan-L) and, subjects not controlled with GLP-1RAs (LixiLan-G*. While, in SoliMix trial, *participants with suboptimally controlled BI–treated T2DM* were evaluated [26-34].

The baseline (BL) characteristics, objectives, inclusion/exclusion criteria and background treatment of the participants in the LixiLan program and in the SoliMix trial are summarized in Table 1.

**Table 1.** Baseline characteristics and background treatment of the participants in the LixiLan program and the SoliMix trial

Trial, (blinding, Rn) (Ref)	LixiLan-L (O-L, 1:1) (26)	LixiLan-O (O-L, 2:1:1) (27)	LixiLan-G (O-L, 1:1) (28,29)	SoliMix (O-L, 1:1) (34)
**Primary objectives**	To demonstrate the superiority of iGlarLixi to iGlar in A1c change	To compare iGlarLixi to Lixi alone and to iGlar alone (with Met) in A1c change	To demonstrate the superiority of iGlarLixi to GLP-1 RA in A1c change	To show that iGlarLixi is non-inferior to BIAsp 30 (A1c reduction) or superior in body weight change
**Patients (adults)**	Inadequate glycemic control, despite the use of a BI, with ≥1 OADs	Inadequate glycemic control despite treatment with Met with ≥1 OADs	Daily or weekly use of GLP- 1RA therapy	Inadequate control with BI (with ≥1 OADs)
**Exclusion criteria**	Renal, hepatic, or cardiovascular conditions, uncontrolled hypertension, cancer, a histor y of medullar y thyroid carcinoma or elevated calcitonin levels, a histor y of pancreatitis, or amylase and/or lipase levels >3 times the upper limit of the normal laborator y range			Any DM (other than T2D); use of OADs (other than BI, Met or SGLT2i); use of weight-loss drugs
**Therapy at screening**	Met ± OADs	Met ± OADs	GLP-1RA + Met ± SU ± Pio	BI + Met alone or Met combined + SGLT2i
**iGlarLixi (presentation)**	iGlarLixi was available in 2 different 3 mL prefilled pens, one containing 100 U of iGlar and 50 μg of Lixi/mL, the other pen containing 100 U of iGlar and 30 μg of Lixi/mL			
**SMPG goals**	80-100	80-100	80-100	80-110
**A1c (%)**	≥7.5% to ≤10%	≥7% to ≤9.0% for treated with Met and other OAD; and ≥7.5% to ≤10% for treated with Met alone	7.0-9.0	≥7.5% to ≤10%
**Hypoglycemia categories**	Documented symptomatic hypoglycemia (≤70 mg/dL) and severe symptomatic hypoglycemia (event requiring assistance of another person to actively administer carbohydrate, glucagon, or other resuscitative actions)			
**BMI (kg/m2)**	≤40	≤40	≤40	>20 and ≤40
**Concomitant therapy with OADs**	Met	Met	Met ± Pio ± SGLT2i	Met ± SGLT2i
**Main phase (duration)**	30 weeks	30 weeks	26 weeks	26 weeks
**Extension phase (duration)**	NA	NA	26 weeks	NA

Legend: A1c: glycosylated hemoglobin; BI: basal insulin; BIAsp 30: Biphasic aspart insulin 30 (30% insulin aspart and 70% insulin aspart protamine); BMI: body mass index;

FPG: fasting plasma glucose; GLP-1RA: GLP-1 receptor agonists; iGlar: glargine U-100; iGlarLixi: glargine + lixisenatide (Lixi); kg: kilograms; Met: metformin;

NA: not applicable; Pio: pioglitazone; OADs: oral anti-diabetics; O-L: open-label; Ref: reference; Rn: randomization; SGLT2i: SGLT2 inhibitor; SMPG: self-measured plasma glucose;

SU: sulphonylurea.

## 3. Results

In the LixiLan-O trial, iGlarLixi was found to complement the individual effects of iGlar and Lixi to achieve significant reductions in A1c, with no significant increase in risk of hypoglycemia or weight gain (compared to iGlar), and with few gastrointestinal (GI) adverse events (AEs), when compared to Lixi.

In the LixiLan-L, a high proportion of individuals receiving iGlarLixi achieved glycemic control, with a beneficial effect on body weight, without a significant increase in the risk of hypoglycemia.

In the LixiLan-G trial, iGlarLixi significantly improved glycemic control without documented symptomatic hypoglycemia. The rate of nausea and vomiting and the number of documented symptomatic hypoglycemic events was low overall, but occurred to a greater extent with iGlarLixi. These results were consistent and sustained over an extension period (week 52); for example, the average A1c of 6.7% at week 26 was maintained at week 52; additionally, iGlarLixi was also associated with significant and sustained reductions in FPG and PPG.

Moreover, in the LixiLan trials, the risk of hypoglycemia was higher in participants who received iGlar (either as a monocomponent or in combination), although in general, the hypoglycemic episodes were low in the LixiLan-O and LixiLan-G trials (when compared to the LixiLan-L trial). GI AEs were more frequent in individuals receiving GLP-1RA (relative to those receiving iGlarLixi or those receiving iGlar), although the rate was similar between treatment groups.

**Figure 1.**
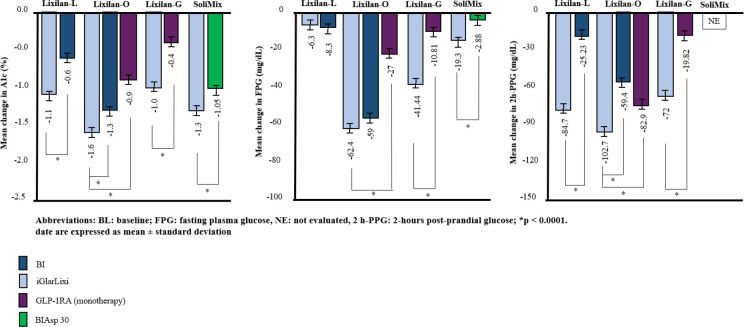
Results of the LixiLan program on: mean changes from BL in (a), A1c (%); (b), fasting plasma glucose (FPG); (c), 2-hours post-prandial glucose (2 h-PPG)

The results of the LixiLan-L, LixiLan-O and LixiLan-G are summarized in the Figure 1 and Table 2.

**Table 2.** Secondary outcomes in the LixiLan program

	LixiLan-L		LixiLan-O			LixiLan-G	
**Interventions (n)**	iGlarLixi (366)	iGlar (365)	iGlarLixi (468)	iGlar (466)	Lixi (233)	iGlarLixi (252)	GLP-1RA (253)
Patients, n (%) reaching A1c <7% at week 30*	201 (54.9%)	108 (29.6%)	345 (74%)	277 (59%)	77 (33%)	156 (61.9%)	65 (25.7%)
**Proportion difference (95% CI) vs GLP-1RA, p-value**	NA		NA			36.1% (28.1% to 44.0%), <0.0001	
**Mean body weight (kg)**							
**BL (mean)**	87.8	87.1	89.4	89.8	90.8	93.01	95.49
**LS change from BL (mean)**	-0.7	0.7	-0.3	1.1	-2.3	1.89	-1.14
**Comparison vs iGlar (95% CI), p-value**	-1.4 (-1.8 to -0.9), <0.0001		-1.4 (-1.9 to -0.9), <0.0001			NA	
**Comparison vs Lixi (95% CI)**	2.01 (1.4 to 2.6)						
**Comparison vs GLP-1RA (95% CI), p-value**	NA		NA			-3.03 (2.417 to 3.643), <0.0001	
Patients, n (%) reaching A1c< 7.0% with no body weight gain at week 30*	125 (34.2%)	49 (13.4%)	202 (43.2%)	117 (25.1%)	65 (27.9%)	NE	NE
**Proportion difference vs iGlar (95% CI), p-value**	20.8 (15.0 to 26.7), <0.0001		18.1 (12.2 to 24.0), <0.0001			NA	
**Proportion difference vs Lixi (95% CI)**	NA		15.2 (8.1 to 22.4)			NA	
**iGlar daily dose (units)**							
**BL (mean)**	35.0	35.2	10 (initial daily dose)	10 (initial daily dose)	NA	NA	NA
**Endpoint (mean)**	46.7	46.7	NA		NA	NA	
**LS insulin dose change at week**							
**30 (mean)**	10.6	10.9	NA		NA	NA	
**LS insulin dose at week 30 (mean)**			39.8	40.5	NA	NA	NA

Legend: A1c: glycosylated hemoglobin; BL: baseline; CI: confidence interval; iGlar: glargine U-100, GLP-1RA: GLP-1 receptor agonists, iGlarLixi: glargine + Lixisenatide (Lixi), kg: kilograms; Met: metormin, NA: not applicable, NE: not specified; Pio: pioglitazone, OADs: oral anti-diabetics, O-L: open-label, SGLT2i: SGLT2 inhibitor, SU: sulphonylurea; vs: versus; *At week 26 in LixiLan-G trial.

In the SoliMix trial, the A1c was reduced by 1.3% with iGlarLixi and 1.1% with BIAsp 30, meeting noninferiority (least squares [LS] mean difference −0.2% [97.5% CI −0.4 to −0.1]; P < 0.001). iGlarLixi was also superior to BIAsp 30 for body weight change (LS mean difference −1.9 kg [95% CI −2.3 to −1.4]) and percentage of participants achieving A1c <7% without weight gain and A1c <7% without weight gain and without hypoglycemia (all P < 0.001).

iGlarLixi was also superior to BIAsp 30 for A1c reduction (P < 0.001) and the incidence and rates of hypoglycemia were lower with iGlarLixi versus BIAsp 30. The percentage of participants who had at least one AE was slightly higher in the iGlarLixi group (32.6%) compared with the BIAsp 30 group (27.7%), the difference being mainly due to the higher incidence of GI AEs in the iGlarLixi group (10.4% versus 2.3%).

### 3.1 FRCs that assess the efficacy and safety of iDegLira

The efficacy and safety of iDegLira was evaluated in nine RCTs (DUAL program), in three different populations of participants with T2DM: *subjects not controlled with OADs (DUAL I, IV, VI, VIII and IX)* [35-39]; *subjects not controlled with GLP-1RAs (DUAL III)* [39]; *subjects not controlled with BI (DUAL II, V and VII)* [41-48].

The BL characteristics, objectives, inclusion and exclusion criteria, and background treatment of the participants in the DUAL program are summarized in the Table 3.

**Table 3.** Baseline characteristics and background treatment of the participants in the DUAL program

Trial (blinding, Rn) (Re.	DUALI (O-L, 2:1:1) (35)	DUAL II (D-B, 1:1) (41)	DUAL III (O-L, 2:1) (40)	DUAL IV (D-B, 2:1) (36)	DUALV (O-L, 1:1) (42)	DUAL VI (O-L, 1:1) (37)	DUAL VII (O-L, 1:1) (43)	DUAL VIII (O-L, 1:1) (38)	DUAL IX (O-L, 1:1) (39)
**Primary objectives**	Change in Ale (iDegLira vs Lira)	Change in Ale (iDegLira vs iDeg)	Change in Ale (iDegLira vs unchanged GLP-IRA)	Change in Ale (iDegLira vs placebo)	Control of Ale (iDegLira vs iGlar	Control of Ale over time (iDegLira lWT vs 2WT)	Change in Ale (iDegLira vs iGlar and iAsp	Durability of treatment effect (iDegLira vs iGlar)	Change in Ale (iDegLira vs iGlar)
**Patients (adults)**	Insulin-nafve uncontrolled on OADs	Uncontrolled on BI + OADs	Uncontrolled on GLP-IRA + OADs	Insulin-nafve uncontrolled on OADs	Uncontrolled on BI t Met	InsulinnaiVe uncontrolled on OADs	Uncontrolled on BI t Met	InsulinnaiVe uncontrolled on OADs	Insulin-naive uncontrolled on OADs
**Exclusion criteria**	Impaired liver or kidney function, calcitonin ≥50 ng/L> history of medullary thyroid carcinoma or multiple endocrine neoplasia type 2, cardiovascular disorders, cerebral stroke, severe uncontrolled treated or untreated hypertension, acute treatment required proliferative retinopathy or maculopathy, history of chronic pancreatitis or idiopathic acute								
**Therapy at screening**	Met ± Pio	BI + MetiSU or Glin	GLP-IRA + MetiSUor Pio	SU + Met	iGlar-100 + Met	Met ± Pio	Glar-1001 Met	Met + Pio + SU + G!in±DPP-4i	SGLT2Í ± Met ± DPP-4Í ± Pio
**iDegLira (presentation)**	1 pen containing lUof iDeg and 0.036 mg of Lira was used								
**SMPG goals**	iDegLira and iGlar doses were titrated with the goal of reaching a target FPG (SMBG) of 72-90 mg/dL (except DUAL IV) where a less aggressive titration was made (72-108 mg/dL). In DUALNI, iAsp doses were titrated in order to reach a preprandial and bedtime glucose goal of 72-108 mg/dL								
**Ale (%)**	7.010.0	7.510.0	7.09.0	7.0-9.0	7.010.0	7.0-10.0	7.0-10.0	7.0-11.0	7.011.0
**Hyjjoglycemia categories**	The episodes of severe and minor hypoglycemia was assumed as confirmed hypoglycemia: the minor hypoglycemic episodes were defined as <56 mg/dL and did not meet criteria for severe hypoglycemia. Severe episodes were defined as one that required the assistance of a third party: nocturnal hypoglycemia was defined as any event occurring be^een 00:01 and 00:59 h								
**BMI (kg/m^2^)**	≤40	≥27	≤40	≤40	≤40	≤40	≤40	≤40	≥20 to ≤40
**Concomitant therapy with OADs**	Met ± Pio	Met	MetiSUiPio	SU + Met	Met	Met ± Pio	Met	Met±Pi0±SU± Glin±DPP-4i	SGLT2Í ± Met ± DPP-4Í ± Pio
**Main phase (duration)**	26 weeks	26 weeks	26 weeks	26 weeks	26 weeks	32 weeks	26 weeks	104 weeks	26 weeks
**Extension phase (duration)**	26 weeks	NA	NA	NA	NA	NA	NA	NA	NA

Legend: A1c: glycosylated hemoglobin; BI: basal insulin; BMI: body mass index; D-B: double bind; DPP-4i: DPP-4 inhibitors; FPG: fasting plasma glucose; Glin: glinide;

GLP-1RA: GLP-1 receptor agonists; iAsp: insulin aspart; iDegLira: degludec + liraglutide (Lira); iGlar: insulin glargine U-100; Met: metformin; NA: not applicable;

OADs: oral anti-diabetics; O-L: open-label; Pio: pioglitazone; Rn: randomization; Ref: reference; SGLT2i: SGLT2 inhibitor; SMPG: self-measured plasma glucose;

SU: sulphonylurea; vs: versus; 1WT: once-weekly titration; 2WT: twice-weekly titration

In DUAL I trial, iDegLira was associated with a reduction in the frequency of AEs associated with its components (weight gain, hypoglycemia, and GI AEs); in DUAL IV, the addition of iDegLira to sulphonylurea (SU) −with or without Met− reduced A1c, but there was an increase in weight and the risk of hypoglycemic events. In DUAL VI, iDegLira treatment demonstrated a similar efficacy and safety profile, with weight loss and a low risk of hypoglycemic events. In DUAL VIII, a greater treatment effect with iDegLira (compared to iGlar) was demonstrated in individuals who did not require intensification of therapy, with a slight increase in weight, and a low frequency of hypoglycemic events. In DUAL IX, the superiority of iDegLira (relative to iGlar) when added to an SGLT2i (with or without other OADs) was confirmed for glycemic control, with no significant changes in weight, with few hypoglycemic events.

In DUAL III, iDegLira was found to be superior (relative to GLP-1RA) in achieving significantly lower A1c and FPG levels; however, weight gain was higher with iDegLira, as was the number of hypoglycemic events.

In DUAL II, V and VII, iDegLira demonstrated superiority in glycemic control over iDeg and iGlar, and demonstrated non-inferiority over the basal-bolus insulin (BBI) regimen. Hypoglycemia rates with iDegLira were numerically lower than iDeg and significantly lower than iGlar (high-dose, titratable) and the BBI regimen. Additionally, weight loss was significantly greater with iDegLira compared to iDeg and iGlar and to the BBI regimen. IDegLira significantly increased the risk of confirmed hypoglycemia (overall and nocturnal) compared to placebo + SU and GLP-1RAs, and also demonstrated a lower risk of overall hypoglycaemia (relative to iDeg). A similar reduction in the risk of global hypoglycemia was also seen in those who had experienced prior insulin management. Moreover, iDegLira decreased the risk of hypoglycemia (severe and nocturnal) in relation to the BBI regimen.

Additionally, the risk of hypoglycemia was higher with iDegLira in participants who received management with SU (DUAL IV) or with SU + BI, and the GI AEs were more frequent in individuals who received GLP-1RAs (in relation to those who received iDegLira or BIs).

**Figure 2.**
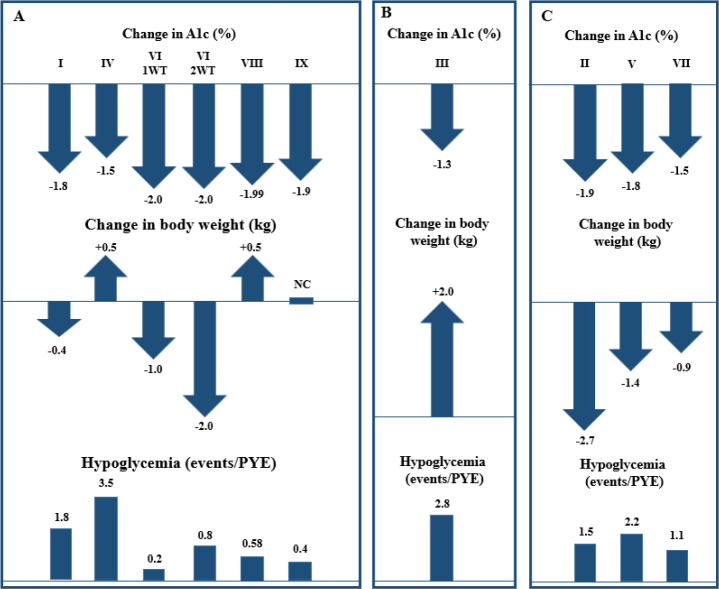
Results of the DUAL program on: changes in A1c value; effects on body weight; and hypoglycemic events in participants with OADs -DUAL I, IV, VI, VIII, and IX- (a); uncontrolled participants with GLP-1RA -DUAL III- (b); and uncontrolled participants with BI -DUAL II, V, and VII- (c) Abbreviations: A1c: glycosylated hemoglobin; PYE: events per patient-year of exposure; 1WT: once-weekly titration; 2WT: twice-weekly titration

The results of the DUAL program on changes in A1c, effects on body weight and hypoglycemic events are summarized in the Table 4 and Figure 2.

**Table 4.** Secondary outcomes in the DUAL program

Trials (n)	Intervention/ comparator	FPG (mg/dL) change from BL	PPG (mg/dL) change from BL	A1c < 7% (% patients)	A1c ≤ 6.5% (% patients)	A1c < 7% (% patients)*
**DUAL I (1,663)**	iDegLira	-65	-44	81	70	36
	iDeg	-65	-37	65	47	14
	Lira	-32	-21	60	41	52
**DUAL II (413)**	iDegLira	-62	NA	60	45	40
	iDeg	-46	NA	23	13	8.5
**DUAL III (438)**	iDegLira	-54	NA	75	63	NA
	GLP -1RA	-11	NA	36	23	NA
**DUAL IV (435)**	iDegLira	-47	NA	79	64	NA
	Placebo	-6	NA	29	12	NA
**DUAL V (557)**	iDegLira	-50	-11.5	72	55	39
	iGlar	-49	-10	47	31	12
**DUAL VI (420)**	iDegLira 1WT	-78	NA	90	84	NA
	iDegLira 2WT	-82	NA	89	85	NA
**DUAL VII (506)**	iDegLira	-43	NA	66	50	35
	iGlar + iAsp	-34	NA	67	45	5
**DUAL VIII (1,012)**	iDegLira (w:26)	-71	NA	79	64	30
	iGlar (w:26)	-68	NA	56	35	9
	iDegLira (w:104)	-71	NA	55.5	43	17
	iGlar (w:104)	-67	NA	28.5	22	5.5
**DUAL IX (420)**	iDegLira	-67	-73	85	75	42
	iGlar	-63	-53	71	49.5	17

Legend: A1c: glycosylated hemoglobin; BL: baseline; FPG: fasting plasma glucose; GLP-1RA: GLP-1 receptor agonists; iAsp: insulin aspart; iDegLira: Degludec/Liraglutide (Lira); iGlar: insulin glargine U-100; iGlarLixi: iGlarLixi: insulin glargine + Lixisenatide (Lixi), NA: not applicable; PPG: postprandial glucose, w: week; 1WT: once-weekly titration; 2WT: twice-weekly titration; * Without hypoglycemias/weight gain

### 3.2 Summary of the efficacy and safety of iGlarLixi and iDegLira in T2DM

Both FRCs show significant reductions in the value of A1c, FPG and PPG (the reduction in PPG was mainly observed with iGlarLixi), the risk of hypoglycemia is lower with FRCs compared to BIs (but the risk is higher with FRCs, compared to the use of GLP-1RAs); GI AEs are similar between the different groups, and tend to attenuate over time and with adequate dose titration with FRCs. Insulin doses are lower in subjects receiving FRCs and weight gain is evidently lower in subjects receiving FRCs (compared to those using different insulin therapy schemes). The summary of different outcomes such as: final dose of insulin; documented, severe, and nocturnal hypoglycemia, trends in body weight, and different AEs related to both FRC is shown in Tables 5 and 6.

**Table 5.** Summary of outcomes in the DUAL and LixiLan trials (final dose of insulin, hypoglycemia and trend in body weight)

Trials	Inter vention/ Comparator	Mean insulin dose at last recorded visit (units)	Documented hypoglycemia (% patients)	Severe hypoglycemia (% patients)	Nocturnal hypoglycemia (% patients)	Weight trend (kg)
**DUAL I**	iDegLira	38 (13)	32	0.36	NA	-0.5
	iDeg	53 (28)	39	0.48	NA	+1.6
	Lira	-	7	-	NA	-3
**DUAL II**	iDegLira	45	24	0.5	6	-2.7
	iDeg	45	25	0	8.5	0
**DUAL III**	iDegLira	33.5	32	0.3	11	+2
	GLP-1RA	-	3	0	0.7	-0.8
**DUAL IV**	iDegLira	28	42	0.7	12	+0.5
	Placebo	-	17	0	7	-1
**DUAL V**	iDegLira	41	28	0	6	-1.4
	iGlar	66	49	0.4	24	+1.8
**DUAL VI**	iDegLira 1WT	41	9	0	2	-1
	iDegLira 2WT	41	24	0.5	7	-2
**DUAL VII**	iDegLira	40	20	1.2	5	-0.9
	iGlar + iAsp	84	53	1.6	19	2.6
**DUAL VIII**	iDegLira	37	NA	NA	NA	+1.7
	iGlar	52		NA	NA	+3.4
**DUAL IX**	iDegLira	36	13	NA	2.9	0
	iGlar	53.5	19.5	NA	6.2	+2
**LixiLan-L**	iGlarLixi	47	40	1.1	NA	-0.7
	iGlar	47	42.5	0.3	NA	+0.7
**LixiLan-O**	iGlarLixi	39.8	26	0	NA	-0.3
	iGlar	40.3	24	0.2	NA	+1.1
	Lixi	-	6.4	0	NA	-2.3
**LixiLan-G**	iGlarLixi	43.5	28	9.4	NA	+1.9
	GLP-1RA	-	2.3	0.4	NA	-1.1

Legend: -: absent; GLP-1RA: GLP-1 receptor agonists; iDeg: insulin degludec U-100; iDegLira: Degludec/Liraglutide (Lira); iGlar: insulin glargine U-100; iGlarLixi: insulin glargine + Lixisenatide (Lixi); kg: kilograms; NA: not applicable; 1WT: once-weekly titration; 2WT: twice-weekly titration

**Table 6.** Overview of the adverse events related to the use of iDegLira and iGlarLixi

Outcomes	DUAL I	DUAL II	DUAL III	DUAL IV	DUAL V	DUAL VI (1 WT; 2 WT)	DUAL VII	DUAL VIII	DUAL IX	LixiLan-L	LixiLan-O	LixiLan-G
**Safety analysis set**	825	199	291	288	278	209	252	506	209	365	469	255
**AEs (%)**	63	57.8	65.6	64.2	NR	50	59.1	76	61.7	53.4	56.9	63.9
**Serious AEs (%)**	4.6	3.5	3.1	4.9	1.8	3.3; 6.7	4.8	11.8	2.9	5.5	3.8	3.9
**Severe AEs (%)**	-	-	3.1	4.5	-	NR	2.8	8	2.4	-	-	-
**All-cause mortality (%)**	0.12	-	-	0.3	-	0; 0.4	-	0.4	-	0.3	0.4	-
**Withdrawals (%)**	11.7	16.5	5.5	13.1	10	9; 2.9	0.8	4.3	4.8	8.2	6.2	9.8
**Withdrawals for AEs (%)**	1.3	1	0.3	3.1	3.2	2.9; 1	0.4	3	-	3.3	2.6	3.9
**Withdrawals for non-compliance (%)**	NR	NR	3.1	4.5	NR	-	-	-	-	1.1	1.7	0.8
**Withdrawals for ineffective treatment (%)**	0.1	1	0.7	0.7	NR	-	-	-	-	-	0.2	0.4
**Nausea (%)**	10.3	6.5	3.1	8.7	9.4	5.3; 5.2	11	6.1	5.7	10.4	9.6	8.6
**Diarrhea (%)**	10.2	6.5	4.5	4.2	7.2	NR	6.3	7.7	NR	4.4	9.0	5.5
**Vomiting (%)**	5	NR	NR	2.4	5	NR	NR	0.2	NR	3.6	3.2	3.1
**Headache (%)**	12.8	6	9.3	5.2	4	NR	5.6	11.5	8.6	5.7	5.1	3.9
**Nasopharyngitis (%)**	13.9	2.5	8.3	8.7	NR	6.2; 4.3	4.8	11.3	7.7	8.7	5.5	9.8
**Increase in lipase levels (%)**	5.8	6	9.9	9.4	NR	NR	NR	-	5.7	NR	0.9	NR
**Decreased appetite (%)**	2.7	NR	NR	NR	NR	NR	NR	NR	NR	NR	NR	NR
**Confirmed pancreatitis (n)**	1	NR	NR	-	-	-	-	-	-	-	-	NR
**Thyroid neoplasm (n)**	-	NR	NR	-	-	-	-	-	-	-	-	1

Legend: -: absent; AEs adverse events: NR: not reported; 1WT: once-weekly titration; 2WT: twice-weekly titration

## 4. Discussion

Clinically, the therapeutic failure to the OADs is common, and the intensification of management and achievement of glycemic control goals have been associated with a reduction in various micro and macrovascular outcomes. In addition to the intensification with BI, combined regimens with rapid-acting insulins or GLP-1RA appear; in fact, recent management guidelines recommend the combined use of BI + GLP1RA in individuals with an A1c value ≥10% or with an A1c value >2% above the individualized goal for the patient, since in individuals in where there is a failure of β-cell insulin secretion, management with GLP-1RAs monotherapy may not be effective (especially in controlling FPG) [49-53].

Certain recommendations must be taken into account for the use of FRCs, for instance, most OADs can be administered with FRCs, except the DPP-4i (there is no evidence in favor of the use of DPP-4i + FRCs in relation to its effectiveness); other OADs, such as pioglitazone (Pio), should be used with great caution in individuals with heart failure, edematous states, and those in whom weight loss is a therapeutic goal. The use of SU + FRC is also possible, taking great care when indicating it in patients with frequent hypoglycemic symptoms or in those who require weight loss, so it may be necessary to reduce the dose of SU.

**Figure 3.**
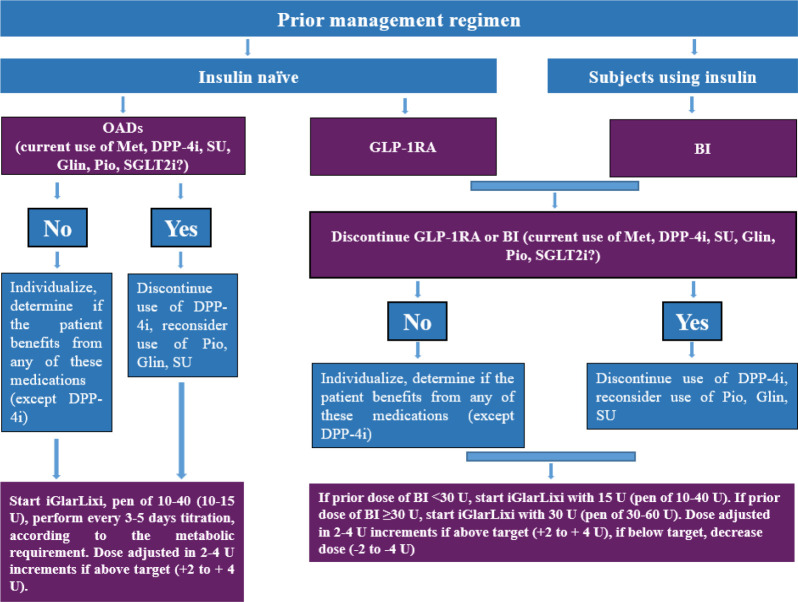
Recommendation algorithm for starting iGlarLixi Abbreviations: BI: basal insulin; DPP-4i: dipeptidyl peptidase-4 inhibitors; Glin: glinides; GLP-1-1RA: GLP-1 receptor agonist; Pio: Pioglitazone; SGLT2i: sodium-glucose cotransporter 2 inhibitors; SU: Sulphonylurea

In relation to the starting doses of both FRCs, iGlarLixi should be administered once daily (OD) and due to the known impact on PPG (mediated by Lixi), it is recommended to administer during the 60 minutes before any of the three main meals. The availability of two commercial presentations for iGlarLixi allows, in those individuals receiving OADs or GLP-1RA, to start with low doses; while, in patients receiving treatment with BI, it should be taken into account always the dose that is received (Figure 3). Both presentations at the top of the dose (40 or 60 U/day of insulin) provide 20 µg of Lixi, and allow its use in a significant proportion of individuals who need high doses of BI (for the presentation of 30-60 U).

**Figure 4.**
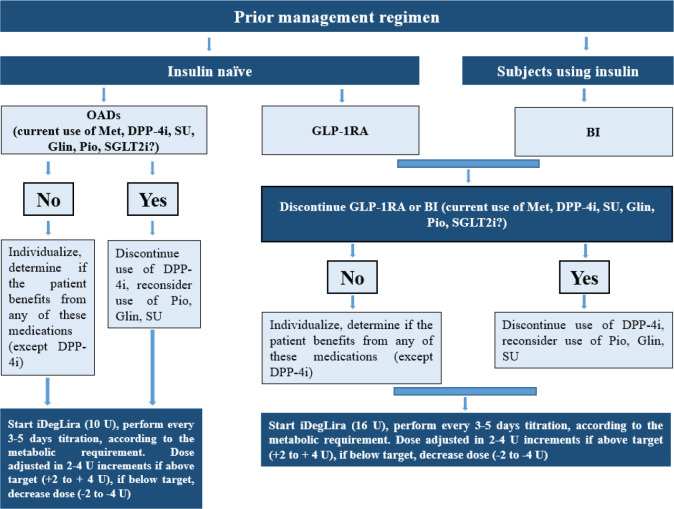
Recommendation algorithm for starting iDegLira Abbreviations: BI: basal insulin; DPP-4i: dipeptidyl peptidase-4 inhibitors; Glin: glinides; GLP-1-1RA: GLP-1 receptor agonist; Pio: Pioglitazone; SGLT2i: sodium-glucose cotransporter 2 inhibitors; SU: Sulphonylurea

Otherwise, starting doses of iDegLira (OD) in patients receiving OADs are low, while in individuals managed with GLP-1RA or BI, the dose is slightly higher (both doses according to the protocol of DUAL trials) (Figure 4). Some concern may arise with the starting dose in patients taking high-dose BI; however, in the DUAL trials, regardless of the BI dose prior to randomization, iDegLira was found to be effective. Moreover, more than 60% of participants in the DUAL trials who received iDegLira achieved an A1c goal of <7.0% with average doses <50 U/day.

Other considerations must be taken into account when considering FRCs management, especially in individuals with high cardiovascular risk; for example, for the particular case with Lixi, it was shown that it is a “neutral” molecule in relation to the cardiovascular outcomes; while, Lira has shown its beneficial effect in reducing cardiovascular outcomes, this effect is achieved with a dose of 1.8 mg/day [54-56].

Therefore, when considering the use of iDegLira in T2DM individuals with high cardiovascular risk, it should be remembered that in order to deliver a 1.8 mg dose of Lira, 50 U of iDeg are concomitantly received; therefore, in these cases, if the use of BI + Lira is required, it is recommended to use each of its components separately.

Additionally, starting with low doses of FRC is likely to result in a lower rate of GI AEs, which may be very common (due to GLP-1RA), gradual titration to the optimal dose it may be a factor that delays the achievement of glycemic control goals [20,22].

Moreover, to our knowledge, there are no head-to-head comparisons between iGlarLixi and iDegLira to date, however, a systematic review meta-analysis and an indirect comparison have evaluated the efficacy and AEs of both FRCs [57,58].

In the meta-analyses, glycemic control of iGlarLixi or iDegLira was significantly lower (from BL); the comparison between both groups of treatment strategies did not show significant differences in outcomes such as the absolute change in the value of A1c or in body weight. In this meta-analysis, the medications that the participants received were not taken into account, additionally, a high heterogeneity was found between the trials; there were also differences in study designs, for example, the LixiLan trials had a run-in period (while the DUAL trials did not), as well as BL FPG values and control goals glycemic levels differed between the studies, these considerations would allow us to state that, in this meta-analyses, the results obtained are not balanced or reliable.

Similarly, the study that performed the indirect comparison between the two FRCs found that, among individuals with T2DM not controlled with BI, treatment with IDegLira resulted in a greater reduction in A1c, with a greater reduction in body weight (at BL) versus iGlarLixi; however, in this analysis certain assumptions of “equality” were made between the trials, for instance, A1c value, prior use of SU, definitions of hypoglycemia, the presence or absence of a run-in period, the type of design and the duration of the trials, inter alia. With these results, the direction and magnitude of the potential differences that may be found are difficult to predict and obviously place a great limitation on the interpretation of findings (especially by the assumption that all prognostic factors and confounding variables were balanced between trials).

Finally, a systematic review of RCTs was recently published, comparing iGlarLixi versus iDeg + Aspart (iDegAsp), finding that the A1c change with iGlarLixi exceeded that for IDegAsp, the A1c target achievement was greater for iGlarLixi. In addition, the analyzes showed differences in mean postprandial SMPG, which were significantly lower with iGlarLixi. Bodyweight change was more favourable for iGlarLixi, however, the comparisons of hypoglycemia were inconclusive owing to differences in definitions between studies. AEs were more frequent with iGlarLixi because of GI intolerance [59].

Some limitations can be identified in this review, for example, we do not involve real-life trials or post-hoc trials, nor indirect analyzes between molecules in the analysis. It is clear that the real-life trials provide important information on the behavior of different management strategies on the clinical set, under real-life conditions, and complement the information provided by the RCTs. In fact, the real-life trials with both FRCs have been shown to be consistent with the results described in the RCTs [60-66].

Additionally, we only analyzed the “pivotal” trials of iDegLira and iGlarLixi, therefore, we did not evaluate trials carried out in other geographical areas; therefore, the results described here should only be extrapolated to individuals or a population of individuals with inclusion criteria similar to those chosen in the DUAL and LixiLan or SoliMix trials.

### 4.1 Summary

In T2DM, FRCs have been shown to be effective in lowering A1c levels and achieving FPG and PPG targets in various clinical settings; insulin doses with both FRCs were similar to those of individuals receiving insulin management. GI AEs were lower with the FRCs (compared to those receiving GLP-1RA); there was also a significant benefit in favor of FRCs in relation to weight loss and risk of hypoglycaemia (compared to those receiving insulin management).

## Abbreviations

AEs: Adverse events

BBI: Basal-bolus insulin

BI: Basal insulin

BIAsp: 30 Biphasic aspart insulin 30 (30% insulin aspart and 70% insulin aspart protamine)

BL: Baseline

BMI: Body mass index

CI: Confidence interval

DM: Diabetes mellitus

DPP-4i: Dipeptidyl peptidase-4 inhibitors

eGFR: Estimated glomerular filtration rate

ETD: Estimated treatment differences

FPG: Fasting plasma glucose

FRC: Fixed-ratio combinations

GI: Gastrointestinal

Glin: Glinides

GLP-1-1RA: GLP-1 receptor agonist

iAsp: Insulin aspart

iDeg: Insulin degludec U-100

iDegLira: Insulin degludec U-100 + liraglutide

iGlar: Insulin glargine U-100

iGlarLixi: Insulin glargine U-100 + lixisenatide

Lira: Liraglutide

LS: Least-squared

Lixi: Lixisenatide

Met: Metformin

NA: Not applicable or not available

NR: Not reported

NS: Not significant

OADs: Oral antidiabetics

OD: Once daily

O-L: Open-label

Pio: Pioglitazone

PPG: Postprandial glucose

PYE: Patient-year of exposure

RCTs: Randomized clinical trials

SGLT2i: Sodium-glucose cotransporter 2 inhibitors

SMBG: Self-monitoring of blood glucose

SU: Sulphonylurea

T2DM: Type 2 Diabetes mellitus

U: Units

WT: Weekly titration
